# Identification of plasma proteins binding oxidized phospholipids using pull-down proteomics and OxLDL masking assay‡

**DOI:** 10.1016/j.jlr.2024.100704

**Published:** 2024-11-19

**Authors:** Philipp Jokesch, Lisa Holzer, Lydia Jantscher, Sebastian Guttzeit, Rudolf Übelhart, Olga Oskolkova, Valery Bochkov, Bernd Gesslbauer

**Affiliations:** 1 Department of Pharmaceutical Chemistry, Institute of Pharmaceutical Sciences, University of Graz, Graz, Austria; 2 Vanudis GmbH, Heidelberg, Germany; 3 Field of Excellence BioHealth - University of Graz, Graz, Austria

**Keywords:** oxidized lipids, phospholipids/metabolism, LDL/Oxidation/antioxidants, phospholipases, antibodies, glycosaminoglycan, OxPL defense, oxidation specific epitopes

## Abstract

Oxidized phospholipids (OxPLs) are increasingly recognized as toxic and proinflammatory mediators, which raises interest in the mechanisms of their detoxification. Circulating OxPLs are bound and neutralized by plasma proteins, including both antibodies and non-immunoglobulin proteins. The latter group of proteins is essentially not investigated because only three OxPC-binding plasma proteins are currently known. The goal of this work was to characterize a broad spectrum of plasma proteins selectively binding OxPLs. Using pull-down-proteomic analysis, we found about 150 non-immunoglobulin proteins preferentially binding oxidized 1-palmitoyl-2-arachidonoyl-*sn*-glycero-phosphatidylcholine (OxPAPC) as compared to non-oxidized PAPC. To test if candidate proteins indeed can form a barrier isolating OxPLs from recognition by other proteins, we applied an immune masking assay. Oxidized LDL (OxLDL) immobilized in multiwell plates was used as a carrier of OxPLs, while mAbs recognizing OxPC or OxPE were used as “detectors” showing if OxPLs on the surface of OxLDL are physically accessible to external binding partners. Using an orthogonal combination of pull-down and masking assays we confirmed that previously described OxPL-binding proteins (non-fractionated IgM, CFH, and Apo-M) indeed can bind to and mask OxPC and OxPE on liposomes and OxLDL. Furthermore, we identified additional plasma proteins selectively binding and masking OxPC including Apo-D, Apo-H, pulmonary surfactant-associated protein B, and antithrombin-III. We hypothesize that in addition to circulating antibodies, multiple non-immunoglobulin plasma proteins can also bind OxPLs and modulate their recognition by innate and adaptive immunity.

Oxidation of polyunsaturated fatty acids by enzymatic and nonenzymatic mechanisms generates oxylipins, which are found in cells, extracellular matrix, and circulation. A large proportion of oxylipins circulates in blood plasma bound to the phospholipid scaffold (oxidized phospholipids, OxPLs) (1). Active investigation of biologically active OxPLs started after the identification of these lipids as the primary pro-inflammatory and atherogenic components of oxidized LDL (OxLDL) (2). As a result of multiple studies that have confirmed and expanded these findings, OxPLs are currently regarded as pleiotropic lipid mediators exhibiting a variety of biological activities (3). OxPLs are thought to play a role in inflammation (4, 5, 6), atherosclerosis (7), cell death and survival (8, 9), blood coagulation and thrombosis (10, 11), lung endothelial barrier regulation (12), angiogenesis (13), vascular aging (14), and other processes. A causative pathogenic role of endogenously generated OxPLs has been confirmed in animal models of atherosclerosis (15), nonalcoholic fatty liver disease (16), liver fibrosis (17), ischemia–reperfusion injury (18), and osteoporosis (19).

The rapidly growing body of data on the biological significance of OxPLs highlights the necessity for a better understanding of the mechanisms of their neutralization and clearance. Similar to other products of PUFA peroxidation, such as 4-hydroxy-2-nonenal (HNE), malondialdehyde (MDA), malondialdehyde-acetaldehyde (MAA) and 2-(ω-carboxyethyl) pyrrole, OxPLs can form oxidation-specific epitopes (OSE) that are targets of natural antibodies recognizing these small molecules in a free form and covalently bound to proteins (20). OSE are proinflammatory molecules (2, 20, 21, 22, 23, 24) and it is thought that opsonization by natural IgM antibodies facilitates clearance of OSE-containing cellular debris and neutralizes their pro-inflammatory properties, thereby contributing to tissue homeostasis and protection against atherosclerosis (25, 26).

In addition to antibodies, free and protein-conjugated OSEs are removed and inactivated by pattern recognition receptors (20, 27), enzymes (Platelet-activating factor acetylhydrolase (PAF-AH/Lp-PLA2) (28)), and antibody-independent binding by plasma proteins (29). Several circulating proteins capable of binding OSEs have been identified including C-reactive protein (CRP) (30), complement factor C1q (C1q) (31), complement factor (anaphylotoxin) C3a (32), complement factor H (CFH) (33, 34, 35) and complement factor H-related proteins (CFHR) (33, 36, 37, 38). The majority of known OSE-binding proteins interact with OxLDL (30, 31, 34) or MDA/MAA protein adducts (32, 33, 35, 39). Binding of these proteins inhibits proinflammatory effects of OSE and reduces complement activation (35).

Non-immunoglobulin plasma proteins capable of opsonizing OxPLs are less investigated as compared to other OSEs. Direct binding to OxPLs has been shown for CRP (30), CFH (34) and HDL-associated apolipoprotein M (40). In these publications, candidate proteins (CRP, CFH and Apo-M) have been pre-selected based either on their known binding properties or genetic association. Therefore, these studies had no power to identify additional circulating proteins binding to OxPLs. Other studies describing binding partners of OxPLs focused on adducts with (intra)cellular proteins (41, 42, 43, 44, 45) or covalent adducts with plasma apolipoproteins (46). Thus, to the best of our knowledge, there are no studies analyzing a broad spectrum of circulating proteins that can bind OxPLs either covalently or non-covalently. In this work, we performed an unbiased search for circulating OxPL-binding proteins. To this end, we applied pull-down proteomic analysis of plasma proteins using liposomes containing OxPAPC, as well as OxPAPC immobilized in ELISA-type multiwell dishes. We have found that in addition to immunoglobulins, also multiple non-immunoglobulin proteins demonstrated higher binding to OxPAPC as compared to non-oxidized PAPC. The ability of plasma proteins to bind OxPLs and prevent their interaction with other proteins was tested using an immune test by masking OxLDL from recognition by monoclonal antibodies specific for OxPLs. Importantly, newly identified OxPAPC-binding proteins at physiological concentrations demonstrated masking activity comparable to or exceeding that of already identified OxPL-binding proteins CFH and Apo-M. Our data support the notion that non-immunoglobulin plasma proteins, in parallel with other mechanisms, can play a significant role in the inactivation of OSEs.

## Materials and Methods

### Materials

Unless otherwise specified, reagents and chemicals were purchased from Sigma-Aldrich. Lipids were purchased from Avanti Polar Lipids. Gradient grade solvents for LC-MS/MS were purchased from VWR. Pooled human IgM was obtained from Sigma-Aldrich. Mouse monoclonal antibody clone 509 (mAb 509) was prepared by hybridoma technology as previously described (47). Mouse anti-oxidized phospholipid monoclonal IgM (mAb E06), was purchased from Szabo Scandic. HS and DS were purchased from Celsus Laboratories. IgM purified from human serum was purchased from Sigma-Aldrich (I8260).

### Oxidation of PAPC

PAPC was oxidized by exposure of dry phospholipid to air as described (2). The degree of oxidation was monitored by nano-ESI MS (LTQ-XL) in positive-ion mode.

### Human plasma

Pooled human EDTA blood plasma was purchased from Biowest. Fresh plasma samples were collected from multiple individuals using K_3_EDTA blood collection tubes (Vacuette, Greiner Bio-One). Samples were pooled, aliquoted, snap-frozen in liquid nitrogen, and stored at −80°C until use.

### Liposome generation

Biotinylated liposomes containing OxPAPC or PAPC were prepared at a total lipid concentration of 200 μM using thin-film hydration by mixing 90 mol% target lipids with 10 mol% 16:0 biotinylated PE (Avanti Polar Lipids) in 100-mL flasks. The lipid film was formed by evaporating CHCl₃:MeOH (65:35 v/v) at 50°C and 130 rpm for 60 min, followed by overnight desiccation over SiO₂. Lipid films were then hydrated in PBSE containing butylated hydroxytoluene (BHT, 0.01% w/w) at 45°C and 220 rpm for 30 min using glass beads. The resulting mixtures were transferred to 5-mL amber vials and homogenized by sonication for 30 min. Liposome size was measured in UV-cuvettes via dynamic light scattering using a Zetasizer (Malvern Instruments) at 37°C with back-scattering at 137°.

### OxPAPC-liposome pull-down

All liposomes were prepared in triplicate, and the pull-down experiments were performed three times on separate days. 500 μl of PAPC- or OxPAPC-containing biotinylated liposomes were loaded onto spin-columns containing pre-washed (5 × 800 μl PBS; 40 CV) 100 μl Streptavidin Sepharose™ High-Performance beads (17,511,301, Cytiva). As an additional control, 500 μl of PBS was loaded instead of liposomes. To facilitate efficient binding, samples were incubated at 37°C, 300 rpm for 30 min, centrifuged, and flow-throughs reapplied 10 times followed by washing steps (6 × 200 μl PBS; 12 CV). Plasma (200 μl 1:2 diluted in PBS) was added to each column and incubated at 37°C, 300 rpm for 30 min. Samples were centrifuged, and flow-throughs reapplied 10 times prior to extensive washing steps (12 × 800 μl PBS; 96 CV). Proteins bound to the liposomes were eluted stepwise using 100 μl of 8 M urea, 100 μl of 1% (m/v) CHAPS and 100 μl of Laemmli buffer. Between the different elution conditions, beats were washed with 200 μl PBS, to decrease carry-over. Eluates were stored at −20°C until further analysis.

### OxPAPC multiwell plate pull-down

Dried lipids, including OxPAPC, PAPC, and DOPC, were redissolved in ethanol containing 0.01% BHT and 0.5 mM EDTA at a concentration of 100 μg/ml. For immobilization, lipids were transferred to 96-well Nunc-Immuno MaxiSorp™ plates (50 μl/well) and air-dried. 50 μl of plasma (1:5 diluted with phosphate-buffered saline containing 0,5 mM EDTA (PBSE)) was transferred to each well, incubated for 45 min at 37°C followed by extensive washing steps with PBSE. Bound proteins were eluted with Laemmli buffer and separated by SDS-PAGE.

### Heparin affinity chromatography

For depletion of heparin-binding proteins from plasma, 500 μl of heparin-Sepharose (Abcam) was applied to spin columns and washed with 15 x 800 μl PBS. Subsequently, 400 μl plasma (1:4 diluted with PBS) was added to the beads, and the flow-through was collected and reapplied to the beads five times. The resulting final flow-through was used for the masking ELISA. Following this, the beads were washed with 20 × 800 μl PBS (32 CV), and bound proteins were subsequently eluted with 2 × 400 μl 1.5 M NaCl. The eluates were combined and dialyzed against PBSE at 4°C (o/n) using Slide-A-Lyzer® 3500 MWCO dialysis cassettes. Protein concentration was determined with Micro BCA Protein Assay Kit (Thermo Scientific).

For heparin pull-down proteomics, 200 μl of plasma (1:2 diluted with PBS) was applied to spin columns containing 200 μl washed heparin-Sepharose. The flow-through was collected and reapplied five times. The beads were then washed with 18 × 500 μl PBS (45 CV) and 2 × 500 μl 50 mM TEAB containing 137 mM NaCl (5 CV). Proteins were subsequently eluted with 2 × 200 μl 500 mM NaCl in 50 mM TEAB and 2 × 200 μl 2% (m/v) SDS in 50 mM TEAB. For proteomic analysis, the eluates were directly subjected to reduction, alkylation, and digestion using s-Trap columns (see sample preparation for mass spectrometry).

### Sample preparation for mass spectrometry

The eluates obtained from OxPAPC-liposome pull-downs and heparin pull-downs were processed using S-Trap™ mini spin columns (ProtiFi) following the manufacturer’s protocol. In brief, proteins were reduced (20 mM dithiothreitol), alkylated (40 mM iodoacetamide), and digested with trypsin (1:50 w/w) for 18 h. Eluted peptides were lyophilized and resuspended in 0.1% formic acid. For each LC-MS/MS run, 100 ng of peptides were used.

Protein eluates from OxPAPC well plate pull-downs were subjected to in-gel reduction, alkylation, and digestion followed by LC-MS/MS analysis as previously described (45).

### LC-MS/MS analysis

Peptide analysis from both OxPAPC-liposome pull-downs and heparin pull-downs was performed using liquid chromatography on an Ultimate 3000 RSLCnano System (Thermo Scientific). Peptides were separated with a flow rate of 300 nl/min on a C18 Aurora UHPLC column (25 cm × 75 μm ID, 1.6 μm) with an integrated CaptiveSpray emitter (IonOptics). The mobile phases consisted of (A) 0.1% (v/v) formic acid in water and (B) 0.1% (v/v) formic acid in acetonitrile. The HPLC gradient for separation was 2% B for 6 min, 2%–25% B in 90 min, 25%–40% B in 10 min, 40%–80% B in 10 min, 80% B for 10 min and 2% B for 12 min. The LC system was coupled online to a timsTOF Pro ion mobility mass spectrometer with the CaptiveSpray nano-electrospray ion source (Bruker Daltonics). The mass spectrometer was operated in PASEF mode and spectra were recorded in DIA mode as previously described (48).

Analysis of peptides obtained from OxPAPC multiwell plate pull-downs was performed on an UltiMate 3000 RSLCnano system in line with an LTQ XL mass spectrometer (Thermo Scientific) as previously reported (45).

### Data processing and statistical analysis

TimsTOF DIA-PASEF data was processed with DIA-NN (v 1.8) in library-free mode (49) using the UniProt homo sapiens proteome fasta file (download 2020). Following settings were applied: Precursor charge state, 1 to 4; precursor m/z ranges, 300–1800; fragment m/z ranges, 200–1800; protease, trypsin; maximum number of missed cleavages, 1; peptide length, 7 to 30; fixed modification, cysteine carbamidomethylation; variable modifications, methionine oxidation and N-terminal acetylation; maximum number of variable modifications, 1. Mass accuracy was fixed to 20 ppm for MS1 and MS2. The output was be filtered at 0.01 FDR.

The Label-Free Quantification (LFQ) values were uploaded into MaxQuant’s Perseus software (v1.6.15.0) (50). Protein hits that were detected in just one (out of three) LC-MS/MS replicate runs and which were detected in only one or two (out of three) pull-down experiments were removed. Proteins identified based on a single peptide were also excluded. After log_2_-transformation, the missing values were treated as zeros. From each elution condition, mean values were calculated and background (liposome-free beads) corrected. Resulting hits with negative values were excluded. Significant differences (OxPAPC vs. PAPC) were determined by two-sided *t* test and permutation-based FDR calculation, followed by the generation of volcano plots for graphical representation (FDR = 0.05, s0 = 0.5).

LTQ-XL data was analyzed with Mascot (Matrix Science) and the UniProt Homo sapiens proteome fasta file (download 2020). The results were filtered based on peptide scores ≥30 and a 1% false discovery rate was applied using Mascot. To identify proteins enriched with OxPAPC compared to PAPC the Exponentially Modified Protein Abundance Index (emPAI) score (51) was employed.

GO-term enrichment analysis was performed with g:Profiler (v e111_e.g.58_p18_30541362; g:SCS threshold; adj. *P*-value < 0.05) (52).

### Masking ELISA

The anti-OxPL plasma activity was measured by an indirect ELISA method as previously described (47). PBS containing 0.5 mM EDTA (PBSE) was used for washing steps, as well as for diluting plasma samples, BSA, proteins, and antibodies. Plates were precoated with OxLDL (1.5 μg/ml), blocked with 3% fish gelatin, and then incubated at 37°C, with or without 1:20 diluted plasma, both in the presence of BSA (10 mg/ml). OxPLs were detected using either mAb 509 (1:7) and goat anti-mouse IgM HRP-conjugated antibody (1:2000), or mAb E06 (1 μg/ml) and High Sensitivity Streptavidin-HRP (1:1000). The colorimetric reaction was initiated by addition of OPD (0.5 mg/ml) and terminated with 2 M H_2_SO_4_. Plate readings were performed at 492 nm using the EnSight plate reader (PerkinElmer). Data processing and statistical analysis were performed using Prism v10.3.0 software (GraphPad, CA, USA). Processed plasma samples (Ig-depleted plasma, plasma from heparin-Sepharose flow-through, etc.) were diluted to reach the same protein concentration as 1:20 diluted mock-treated plasma.

### Depletion of IgG, IgM, and IgA from plasma and IgM purification

Plasma was depleted of IgM, IgG, and IgA using anti-human IgM antibody coupled to agarose (Sigma), Protein G Mag Sepharose (Cytiva) and Peptide M agarose (Invitrogen) following the manufacturer`s instructions. Typically, appropriate amounts of diluted plasma, based on reported reference ranges of immunoglobulins (53, 54, 55), were mixed with washed antibody beads and transferred into spin-columns. Flowthroughs were reapplied five times, collected, and stored at 0°C until further use.

For IgM-purification, the bound antibody was washed with 8 × 500 μl PBS followed by elution with 4 × 100 μl 0.2 M glycine-HCl (pH 2.9). The eluates were immediately neutralized with 0.8 M Tris-HCl (pH 8.5), combined, and dialyzed against PBSE at 4°C (o/n) using Slide-A-Lyzer 3500 MWCO dialysis cassettes (Thermo Scientific). Protein concentration was determined with Micro BCA Protein Assay Kit (Thermo Scientific).

### ELISA

HUVECtert cells were incubated with increasing concentrations of OxPAPC in a serum-free medium or in a medium containing 1% pooled human plasma for 6 h, followed by the analysis of IL-8 in supernatants by ELISA as previously described (56).

### Metabolic activity of cells

HUVECtert were incubated with OxPAPC (0–60 μM) either in a serum-free medium, or in the presence of 1% pooled human plasma for 24 h. Metabolic activity was determined after incubation of cells for 24 h using an XTT assay (57).

### Western blotting

Samples were separated by SDS-PAGE and transferred to BioTrace nitrocellulose membrane (Cytiva) at 20 V for 25 min. Membranes were washed with PBST and blocked with 3% BSA in PBST. Immunoglobulins were detected using respective HRP-conjugated antibodies: goat anti-human IgG (H + L) (1:3500; W4031, Promega), goat anti-human IgA (1: 6000; A18781, Invitrogen), and goat anti-human IgM (HC) (1: 4000; A18835, Invitrogen). Signal development with Clarity Western ECL (Bio-Rad) was followed by acquiring images using a ChemiDoc (Bio-Rad). Quantitative band analysis was performed in ImageJ (v1.53).

### SDS-PAGE and silver staining

Proteins were diluted in reducing Laemmli buffer, incubated at 90°C for 5 min and separated using 12% (v/v) self-cast acrylamide tris-glycine gels at 20 mA. Silver-staining was performed according to Shevchenko *et al.* protocol (58).

## Results

### Human plasma attenuates the proinflammatory and toxic effects of OxPLs

**Fig. 1:**
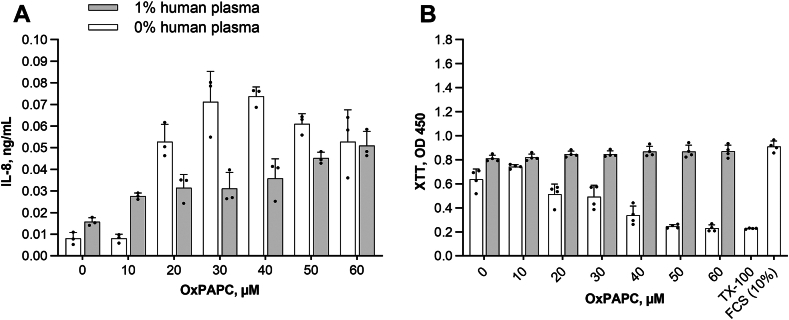
Protective action of human plasma on effects of OxPAPC. A: OxPAPC-induced IL-8 secretion was attenuated by human plasma. HUVECtert cells were incubated with increasing concentrations of OxPAPC in serum-free medium or in medium containing 1% pooled human plasma for 6 h, followed by analysis of IL-8 in supernatants by ELISA. B: Protective effect of human plasma on toxicity of OxPAPC. The cells were incubated with the indicated concentrations of OxPAPC either in serum-free medium, or in the presence of 1% pooled human plasma for 24 h. Metabolic activity of cells was quantified using the XTT assay.

OxPLs have been shown to induce a variety of pro-inflammatory cytokines and chemokines (2, 59, 60) and are traditionally regarded as toxic compounds inducing tissue damage. We found that co-treatment of endothelial cells with OxPAPC and 100-fold diluted human pooled plasma shifted dose dependence of IL-8 induction by OxPAPC to the right (Fig. 1A) and dramatically reduced toxicity of high concentrations of this lipid (Fig. 1B). The data suggest that plasma contains soluble components capable of neutralizing negative effects of OxPLs.

### Identification of plasma proteins binding to OxPAPC and heparin

**Fig. 2:**
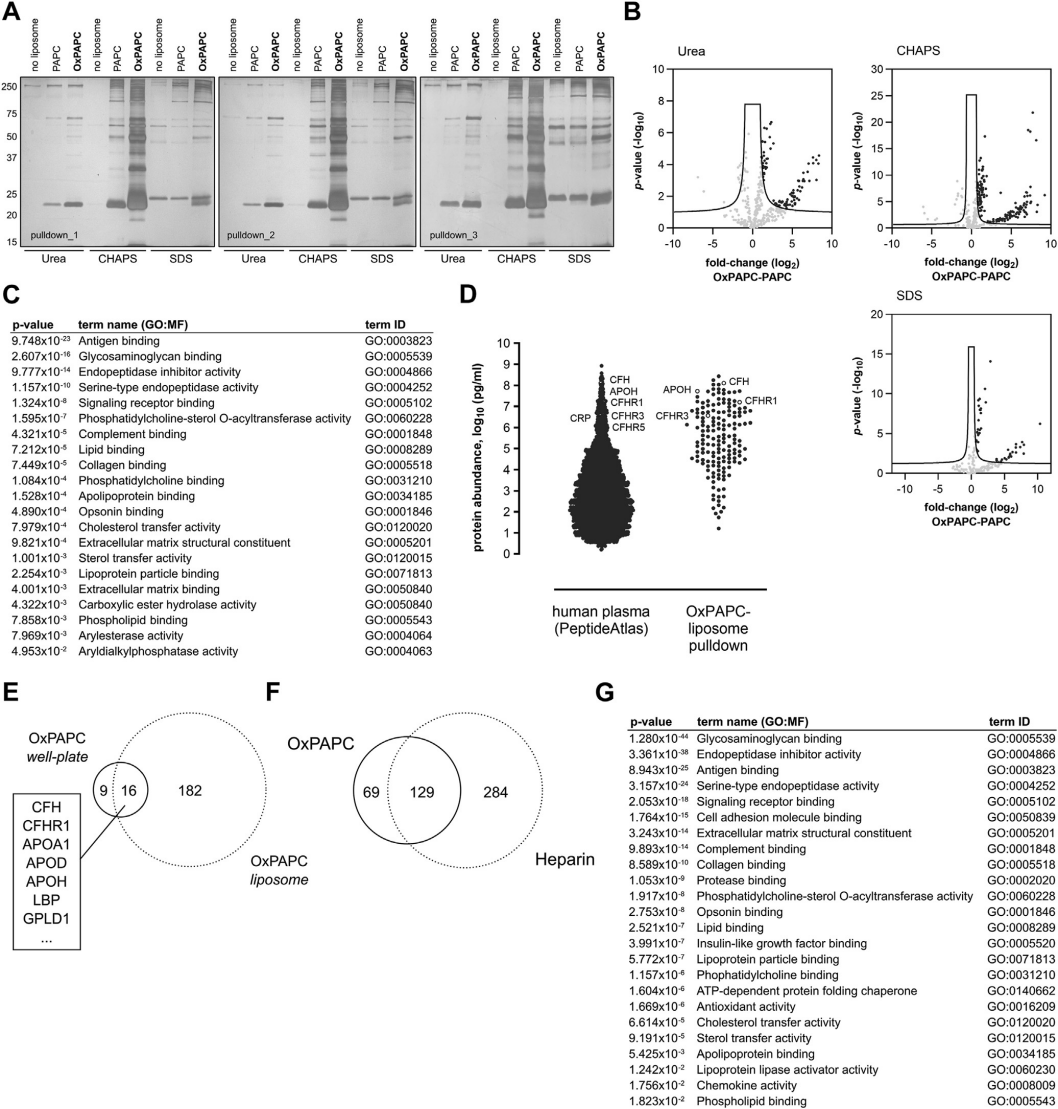
A number of OxPAPC-binding plasma proteins identified by pull-down proteomics also bind to heparin. A: Silver staining of plasma proteins enriched by three independent pull-down experiments using OxPAPC-liposomes, which were incubated with human plasma (diluted 1:2 in PBS) for 30 min at 37°C followed by intensive washes. Captured proteins were eluted consecutively using 8 M Urea, 1% CHAPS and Laemmli buffer. Following tryptic digest, samples were analyzed by LC-MS/MS and DIA-NN. Details of these procedures are described in the “Methods” section. B: Results from 3 independent experiments presented as volcano plots showing log2 fold-change difference (OxPAPC – PAPC) of the three different elution conditions (“UREA”, “CHAPS” and “SDS”) plotted against statistical significance determined by multiple t-tests using Perseus software. FDR = 0.05, s0 = 0.5 (0.2 for SDS). C: Functional enrichment analysis of protein hits from OxPAPC pull-downs. D: Binding to OxPAPC liposomes is not a property of high-abundant proteins. Abundance of proteins enriched via OxPAPC pull-down (right) were mapped against abundance of whole human plasma proteome (left). The dataset was derived from PeptideAtlas. E: Overlap of hits significantly enriched with two formats of pull-down: i) using OxPAPC-liposomes vs. ii) OxPAPC-coated 96-well plates. Technical details are described in the “Methods” section. F: Overlap of protein hits from pull-down using heparin-Sepharose and liposomes containing OxPAPC. G: Functional enrichment analysis of heparin-binding proteins.

To identify OxPL-binding proteins from human blood plasma that may play a role in the inactivation of OxPAPC, we applied pull-down proteomics. Pull-down assays were performed using biotinylated liposomes containing either PAPC or OxPAPC. Liposome-free streptavidin columns were employed as an additional control. Proteins bound to the beads were stepwise eluted using urea, CHAPS, and SDS. Figure 2A illustrates the reproducible enrichment of certain plasma proteins with OxPAPC-containing liposomes compared to PAPC-liposomes and liposome-free beads. Eluted proteins from three pull-down experiments were digested and subjected to LC-MS/MS in triplicates (81 runs in total) followed by protein identification and quantification (DIA-NN) and statistical analysis (Perseus). A protein was regarded to be reliably quantified only if it was detected in all three pull-down experiments (in at least 2 out of 3 LC-MS/MS replicate runs) with at least two peptides. Utilizing three different eluents, we identified 198 plasma proteins significantly enriched with OxPAPC compared to PAPC (Fig. 2B and supplemental File S1). Functional enrichment analysis revealed a variety of molecular functions (GO:MF) among OxPAPC-enriched proteins including antigen binding, glycosaminoglycan binding, phospholipid binding, complement binding, apolipoprotein binding, peptidase inhibitor activity and general “lipid-modifying” enzymatic activities (Fig. 2C). The results of protein functional enrichment analysis are provided in more detail in supplemental File S1. To estimate if the binding of proteins to OxPAPC correlated with their concentration in plasma, the abundance of identified proteins was plotted against the abundance of total human plasma proteome (PeptideAtlas (61)). Despite the identification of several rather high-abundance proteins, our OxPAPC pull-down approach also enriched a considerable number of low-abundance plasma proteins (below 1 μg/ml), which is evidence for certain selectivity of binding (Fig. 2D). Notably, proteins previously reported to interact with OSEs, including CFH, CFH-related proteins, beta-2-glycoprotein 1 (Apo-H) and CRP are high-abundance proteins (Fig. 2D). In order to test if a different aggregation state of OxPLs (liposomes vs. lipid layer on the surface of a multiwell plate) influences their interaction with plasma proteins, we performed adsorption of proteins using ELISA-type plates coated with OxPAPC, PAPC, and DOPC. We observed an enrichment of proteins by OxPAPC compared to PAPC and DOPC (supplemental Fig. S1). Although MS analysis identified fewer proteins preferentially binding to plate-immobilized OxPAPC as compared to the liposome-based approach, most likely due to the lower binding capacity of OxPAPC in the 96-well plates, nearly two-thirds of proteins that bound to OxPAPC on plates were also detected in the liposome-based assay (including CFH, CFHR1, Apo-A1, Apo-D, Apo-H, apo(a), and GPLD1, Fig. 2E and supplemental File S1). While the lipid aggregation state appears to have little effect on protein binding, we cannot entirely rule out the presence of some proteins that preferentially recognize membrane packing defects caused by lipid peroxidation. Furthermore, we compared our identified OxPAPC-binding proteins with the HDL-associated proteome (The HDL Proteome Watch (62)). Approximately 58% of OxPAPC-binding proteins are not contained in this database, providing clear evidence that our approach specifically enriches OxPAPC binders (see supplemental Fig. S7).

Given the significant enrichment of the "glycosaminoglycan binding" GO term in the functional annotation of OxPAPC-binding proteins (Fig. 2C), we aimed to characterize the heparin-binding plasma proteome. Proteins that bound under physiological salt concentrations to heparin-Sepharose, were sequentially eluted with NaCl and SDS, and separated by SDS-PAGE (supplemental Fig. S2). Eluted proteins were reduced, alkylated, and digested using s-trap technology followed by LC-MS/MS. In total, we identified 413 plasma proteins interacting with heparin (supplemental File S1). Remarkably, roughly two-thirds (129 proteins) of OxPAPC-binding proteins were also found to bind to heparin (Fig. 2F). Functional enrichment analysis of heparin-binding proteins showed a significant overlap of molecular functions compared to the OxPAPC-binding proteome (Fig. 2G). Functional enrichment analysis of proteins binding to OxPAPC and heparin, excluding immunoglobulins, showed nearly identical molecular functions as the complete OxPAPC-binding proteome dataset (supplemental Fig. S3). Results of protein functional enrichment analysis of heparin-binding proteins are provided in more detail in supplemental File S1.

These results allow hypothesizing that the structural properties of proteins that are important for their binding to OxPAPC partially overlap with those important for binding to heparin.

### Human plasma exhibits OxLDL-masking activity, a phenomenon that is reversed by heparin

**Fig. 3:**
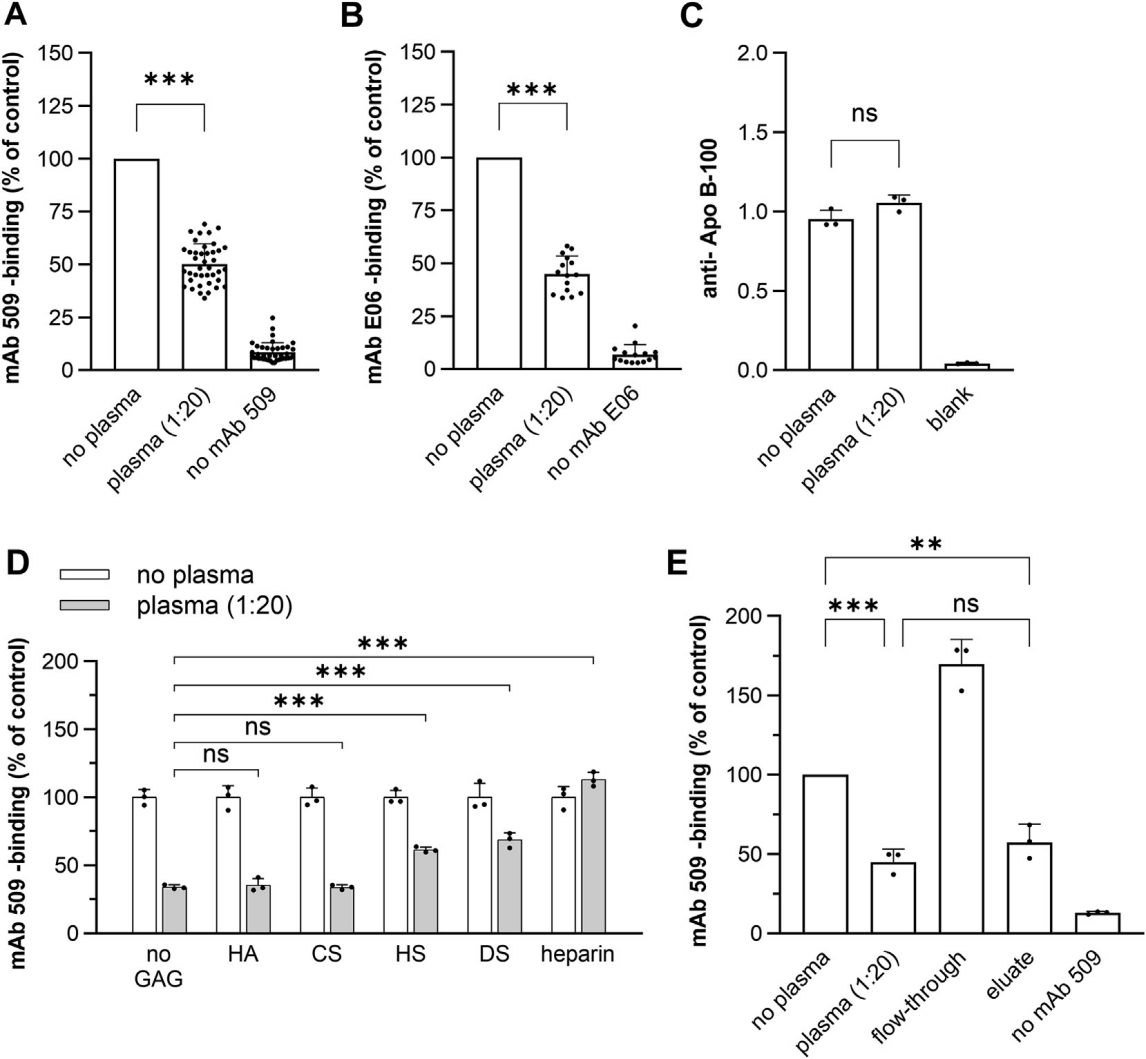
Heparin inhibits the OxLDL-masking activity of human blood plasma. OxLDL-coated 96-well plates were incubated for 1 h at 37°C with PBSE/BSA (10 mg/ml) with or without human plasma (1:20 dilution) and glycosaminoglycans, followed by washing and incubation with mAb 509 and anti-mouse IgM-HRP conjugate. O-phenylendiamine (OPD) and peroxide were used as substrates. Optical density of samples treated without plasma was taken as 100%. ∗*P* < 0.05, ∗∗*P* < 0.01 and ∗∗∗*P* < 0.001. A: Blood plasma pre-treatment reduces interaction of mAb 509 with immobilized OxLDL. The data show ‘OxLDL-masking’-effect measured in triplicates in 40 independent experiments. Statistical significance was determined by one-way ANOVA and Tukey’s post-hoc test. B: Blood plasma pre-treatment reduces interaction of mAb E06 with immobilized OxLDL. The data show ‘OxLDL-masking’-effect measured in triplicates in 15 independent experiments. Statistical significance was determined by one-way ANOVA and Tukey’s post-hoc test. C: Blood plasma treatment does not detach OxLDL from plates. OxLDL immobilized in ELISA plates was detected using anti-apoB100 primary antibody. Statistical significance was determined by one-way ANOVA and Tukey’s post-hoc test. D: Masking activity is inhibited by glycosaminoglycans (GAGs). The inhibitory activity of GAGs correlates with their negative charge and uronic acid contents. All GAGs were applied at 10 μg/ml. Hyaluronic acid (HA), chondroitin sulfate (CS), heparan sulfate (HS), dermatan sulfate (DS) and heparin were dissolved in PBSE/BSA (10 mg/ml) with or without pooled plasma (1:20) and incubated with plates containing immobilized OxLDL. Statistical significance was determined by two-way ANOVA with Tukey’s post-hoc test. E: The OxLDL-masking activity is adsorbed on heparin-Sepharose. Plasma has been separated into heparin-binding and flow-through fractions by chromatography. Note that the flow-through fraction completely lost the OxLDL-masking activity, which was recovered in NaCl-eluate. Statistical significance was determined by one-way ANOVA and Tukey’s post-hoc test.

Previously, we introduced an immune assay to quantify the capability of soluble proteins to interact with OxPCs and OxPEs on the surface of OxLDL (47). We hypothesize that the formation of a protein “barrier” on the surface of OxLDL can prevent the interaction of OSEs with proinflammatory receptors. This assay, referred to as masking assay, is based on monoclonal antibodies mAb 509 (47) and mAb E06 (63), which recognize OxPEs and OxPCs, respectively. Preincubation of OxLDL-coated 96-well plates with diluted pooled human plasma significantly reduced the binding of mAbs 509 and E06 to OxLDL (Fig. 3A, B). Importantly, the reduced binding of mAbs was not due to human serum albumin because all incubations were performed in the presence of 10 mg/ml BSA. This concentration is 10-fold higher than the concentration of human albumin in 1:20 diluted plasma that was used in our experiments. Despite the presence of a high excess of BSA, diluted plasma significantly reduced the binding of mAb 509 and mAb E06 to OxLDL, as compared to preincubation with BSA alone (Fig. 3A, B). Experiments with *human* serum albumin showed no difference in the masking activity compared to *bovine* albumin (data not shown).

To confirm that plasma did not induce detachment of OxLDL from multiwell plates thus leading to reduced mAb 509 binding, we quantified immobilized ApoB100 in the presence and absence of plasma using an ApoB100-specific mAb. Preincubation of OxLDL with plasma did not reduce the amount of immobilized ApoB100 in the wells thus showing that the number of OxLDL particles attached to wells did not change (Fig. 3C).

Because functional enrichment analysis of OxPAPC-binding proteins revealed glycosaminoglycan binding as one of the major hits (Fig. 2C), we next investigated whether heparin and other glycosaminoglycans (GAGs) could influence the masking capacity. To this end, unfractionated heparin, heparan sulfate (HS), dermatan sulfate (HS), chondroitin sulfate (CS), and hyaluronic acid (HA) were added to plasma prior to dilution to the final concentration of 10 μg/ml, equivalent to the concentration of heparin in blood collection tubes. We found that only heparin completely reversed the masking effect of plasma (Fig. 3D). DS and HS partially reduced masking activity, while CS and HA had no effect. Since heparin is highly sulfated and HA is unsulfated, the degree of sulfation is apparently crucial for the reversal of the masking effect. These data point to an important role of electrostatic interactions in the mechanisms that mask OxPLs. Additionally, the uronic acid composition of GAGs seems to influence masking activity. Heparin and DS primarily consist of iduronic acid (IdoA), while HS contains both IdoA and glucuronic acid (GlcA), and CS and HA only contain GlcA. As illustrated in Fig. 3D, only GAGs containing IdoA could reverse the masking activity of plasma.

Heparin could only reverse the masking effect if it came into direct contact with plasma (supplemental Fig. S4B). Preincubation of OxLDL with heparin and its removal before the addition of plasma or the addition of heparin to primary or secondary antibodies did not influence the masking capacity (supplemental Fig. S4A, C). Thus, a direct contact of heparin with plasma proteins was necessary for the reversal of masking activity.

To additionally confirm that the loss of masking activity in the presence of heparin is explained by its binding to plasma proteins but not to immobilized OxLDL, we depleted heparin-binding proteins from plasma using heparin affinity chromatography (supplemental Fig. S5). Plasma depleted from heparin-binding proteins (flow-through) showed no masking activity. In contrast, purified heparin-binding proteins (eluate) demonstrated a masking capacity comparable to mock-treated plasma (Fig. 3E). Importantly, for the masking assay, the heparin flow-through was diluted to the same protein concentration contained in 1:20 diluted mock-treated plasma. The eluate was diluted to reach the same volume as the flow-through. Finally, the flow-through contained 4.08 mg/ml plasma proteins and the eluate contained 0.023 mg/ml plasma proteins, which were directly applied to the masking assay. Thus, the active eluate contained 177-fold *less* total plasma protein than inactive flow-through (plus both contained 10 mg/ml BSA). This is additional evidence that the bulk plasma protein concentration plays minimal role in the masking activity.

To rule out the possibility of heparin leakage from heparin-Sepharose into flow-through fraction thus leading to artifactual masking activity, we applied PBSE to heparin-Sepharose columns and used the resulting flow-through to dilute untreated plasma. Plasma diluted with PBSE passed through heparin-Sepharose showed the same masking activity as plasma diluted with untreated PBSE (supplemental Fig. S4D). Thus, the lack of masking activity in the flow-through could not be explained by the leakage of heparin from the column.

In summary, these findings indicate that the masking activity of certain plasma proteins is inhibited after binding to highly sulfated and IdoA-rich GAGs.

### PAF-AH plays a minor role in the masking activity

**Fig. 4:**
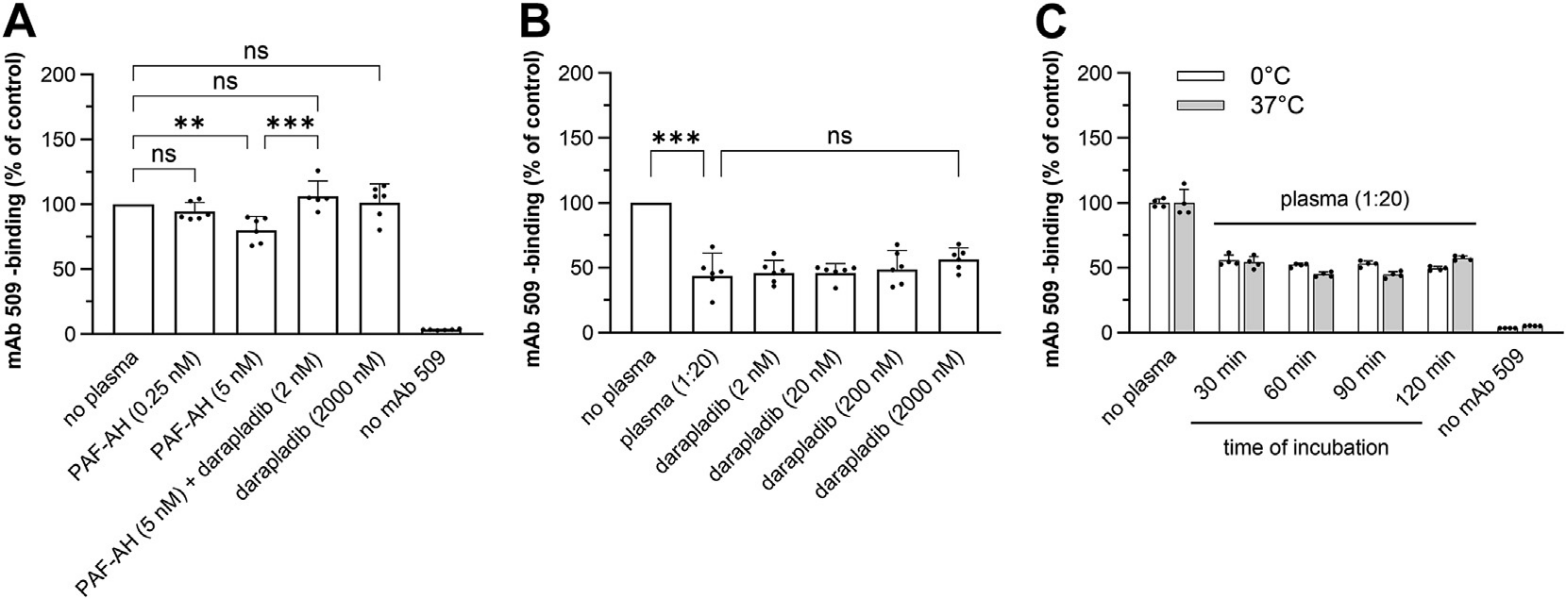
The masking assay is independent on the enzymatic activity of PAF-AH. A: The effect of recombinant PAF-AH in the masking assay was studied according to the standard procedure using incubation with PBSE/BSA (10 mg/ml) as a control or with the same solution containing indicated concentrations of recombinant PAF-AH and its inhibitor darapladib. The data are presented as mean values ± standard deviations (SD), with experiments performed in sextuplicate. Statistical significance was determined by one-way ANOVA and Tukey’s post-hoc test. ∗*P* < 0.05, ∗∗*P* < 0.01 and ∗∗∗*P* < 0.001. B: Inhibition of endogenous PAF-AH has minimal effect on the masking activity of plasma. OxLDL was incubated with plasma (1:20 in PBSE/BSA (10 mg/ml)) either in absence or presence of darapladib. Even at a concentration 400-fold above the reported IC50, darapladib was not able to decrease the masking effect of plasma significantly. C: The masking reaction is minimally influenced by low temperature. The masking assay was performed according to the standard procedure except that incubation of plates with plasma was performed at 37° or 4°C. Data are presented as means ± SD of triplicate measurements. Note that temperature reduction had a minimal or no effect on the masking activity.

Because the plasma enzyme PAF-AH is known to actively degrade OxPLs (64), we investigated the impact of exogenous (recombinant) and endogenous PAF-AH on the masking capacity of plasma. Recombinant PAF-AH exhibited statistically significant masking activity in the absence of plasma at physiological plasma concentrations (5 nM (65)) (Fig. 4A). However, this reduction in mAb 509 binding was not observed when PAF-AH was used at a concentration corresponding to the 1:20 dilution (0.25 nM) of plasma, which was used in our masking assay (Fig. 4A). The masking effect of recombinant PAF-AH could be reversed by the PAF-AH specific inhibitor darapladib. Inhibition of endogenous PAF-AH with darapladib only marginally reversed the masking activity even at very high concentrations of darapladib in plasma which were 400-fold higher than the in vitro reported IC50 (5 nM (66)) (Fig. 4B).

As an additional approach to estimate the impact of enzymatic mechanisms in the masking effect, we tested its temperature dependence. Incubation of immobilized OxLDL with plasma at 4°C to decelerate enzymatic reactions resulted in a minor reduction in the masking effect as compared to 37°C (Fig. 4C). Based on these results, we conclude that PAF-AH and other enzymatic mechanisms play a minor role in the masking assay. Thus, it is likely that the masking assay mainly detects opsonization/physical binding of plasma proteins to OxPLs. This allows to analyze masking capacity of plasma without significant interference from endogenous PAF-AH.

### IgM is the primary immunoglobulin class possessing OxPL-masking activity

**Fig. 5:**
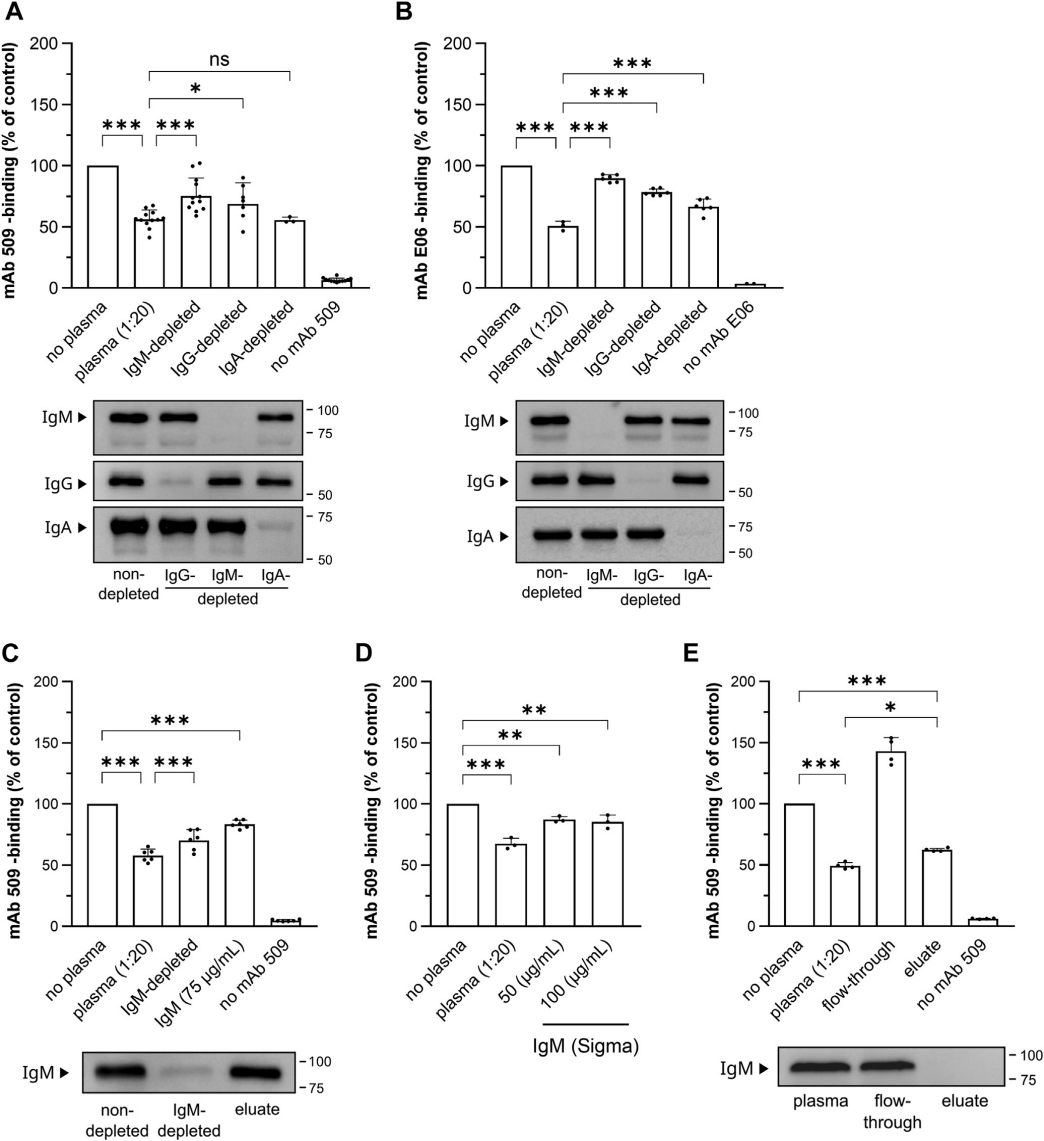
Removal of endogenous immunoglobulins reduces plasma masking capacity against OxLDL recognition by mAbs 509 and E06. A: Effect of IgG-, IgM- or IgA-removal on the masking capacity of blood plasma. mAb 509-masking assay was performed under standard conditions using control plasma or plasma depleted from IgG, IgA and IgM. The data were obtained in 12 independent experiments and are presented as means ± SD. Statistical significance was determined by one-way ANOVA and Tukey’s post hoc test. The Western blot demonstrates the efficiency of the immunoglobulin depletion procedures. B: Experiment with immunoglobulin-depleted plasma samples was performed by the standard masking assay but using mAb E06 recognizing another class of OxPLs (see “Methods”). The data are representative of 5 independent experiments and are presented as means ± SD. Statistical significance was determined by one-way ANOVA and Tukey’s post-hoc test. The Western blot demonstrates the efficiency of the immunoglobulin depletion procedures. C: IgM that was adsorbed on columns, was eluted by changing to acidic pH followed by neutralization. Eluted IgM was tested in a standard mAb 509-masking assay at concentration corresponding to plasma diluted 1:20. Absence of IgM in flow-through and presence in eluate was confirmed by western blotting. D: Masking activity of commercially purchased IgM at concentrations in the range of 20-fold diluted human plasma. Data are presented as means ± SD. Statistical significance was determined by one-way ANOVA and Tukey’s post hoc test. E: Non-immunoglobulin heparin-binding proteins play an important role in the mechanisms of the masking effect. The masking activity data and anti-IgM western blotting show that the eluate from heparin-Sepharose containing heparin-binding proteins demonstrated strong masking activity in spite of the lack of IgM.

Because anti-OxLDL and anti-OxPL autoantibodies are present in the circulation and atherosclerotic plaques (67, 68), we investigated the individual impact of three main Ig classes in the masking activity of plasma. We depleted IgM, IgG or IgA from plasma and compared the activities of depleted plasmas in the masking assay (Fig. 5A, B and supplemental Fig. S6E). Depletion of IgM from plasma resulted in a significant reduction of the masking capacity compared to mock-treated plasma. IgG-depleted plasma showed lower activity, while the removal of IgA from plasma had essentially no effect. In line with mAb 509 data, depletion of IgM from plasma resulted in a significant reduction of masking activity in the assay using mAb E06 (Fig. 5B).

We further tested whether purified IgM possesses masking activity. To this end, we purified IgM from human plasma (supplemental Fig. S6A, B) and investigated the inhibition of mAb 509 binding to OxLDL (Fig. 5C). Purified IgM at a concentration equivalent to 1:20 diluted plasma showed statistically significant masking activity but did not reach masking capacity of mock-treated plasma. In addition, we tested the masking efficacy of commercially available human IgM (Fig. 5D). We observed comparable inhibition of mAb 509 binding to OxLDL with in-house purified (Fig. 5C) and purchased (Fig. 5D) IgM preparations.

In order to exclude the inactivation of IgM as a result of the purification process, non-fractionated plasma was subjected to the same procedures that were applied during IgM purification. No changes in the masking activity of mock-treated plasma as compared to freshly thawed plasma were observed (supplemental Fig. S6C).

In summary, these results demonstrate the importance of circulating antibodies, mainly IgM, for the masking activity of plasma against OxPLs. However, our data suggest that immunoglobulins are necessary but not sufficient for masking. This conclusion is supported by the fact that protein fraction eluted by NaCl from heparin-Sepharose demonstrated high masking activity despite the absence of IgM (Fig. 5E and supplemental Fig. S6D). The data suggest that the mechanism of masking is based on a concerted action of immunoglobulins with additional protein(s) having high affinity to heparin.

### Certain individual plasma proteins exhibit masking activity at physiological concentrations

**Fig. 6:**
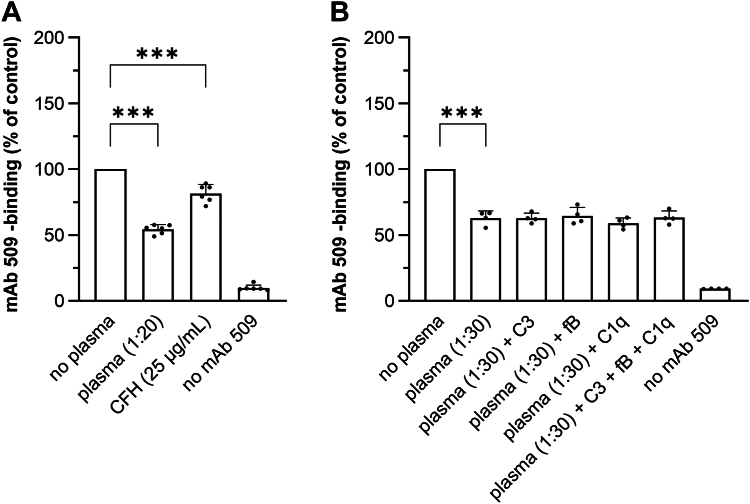
Complement factors C1q, C3 and factor B do not have masking activity. A: Masking assay using mAb 509 was performed according to the standard protocol described in “Methods” using pure complement factor H at 25 μg/ml. B: The masking experiment was performed using factors C1q, factor B and C3 (7, 20 and 130 μg/ml, respectively) alone or in combination. The incubation was performed in the presence of non-saturating dilution of plasma. The presence of low plasma concentration allows to detect i) intrinsic activities of complement factors and ii) combined activities of complement with immunoglobulins in case if complement and immunoglobulins are active only in combination with each other.

Because complement components have been shown to interact with OSEs (29), we tested the masking activity of factors triggering classical and alternative pathways of complement activation, namely factors C1q, factor B and C3. As a positive control for masking activity, we tested another pure complement component that has been previously characterized as an OxPL-binding protein, namely CFH (34). This protein demonstrated moderate but statistically significant masking activity (Fig. 6A). In contrast, factors C1q, factor B, and C3 or their combination were inactive (data not shown). We hypothesized that complement factors may be activated only in the presence of immunoglobulins that bind to OSEs. To test this possibility, we performed a masking assay in the presence of plasma at dilution that induced non-saturating masking effect. Plasma in this case served as a source of immunoglobulins. However, also in the presence of a low concentration of plasma, factors C1q, factor B, and C3 if added individually or as a mixture did not demonstrate any masking activity (Fig. 6B). Because these factors bind to heparin (69), the data suggest that heparin-binding properties alone are not the major determinant of the masking activity.

**Fig. 7:**
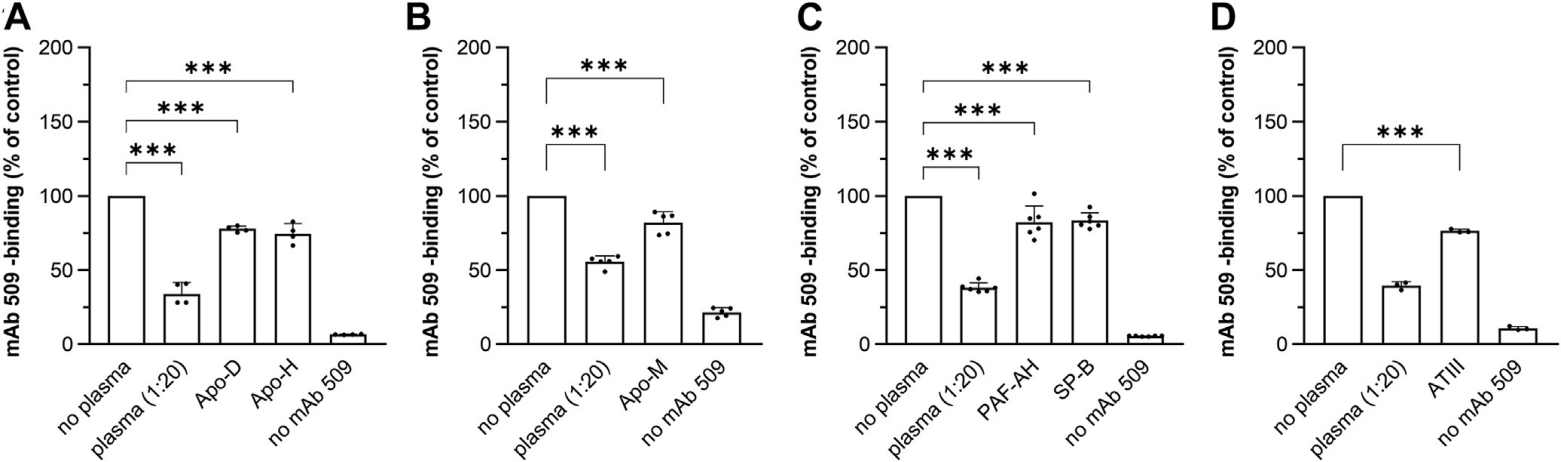
Analysis of selected proteins enriched with OxPAPC-liposome pull-down for mAb 509-masking activity. Several hits from the pull-down experiments that were commercially available were tested in a standard masking assay using mAb 509. Incubation of immobilized OxLDL with recombinant or purified proteins was performed in PBSE/BSA (10 mg/ml) buffer containing physiological concentrations of the following proteins: Apo-D (10 μg/ml), Apo-H (20 μg/ml), Apo-M (5 μg/ml), PAF-AH (0.25 μg/ml), SP-B (5 μg/ml), ATIII (7.5 μg/ml).

We selected from proteomic datasets further candidate proteins for testing in the masking assay. Apolipoprotein D (Apo-D) and Beta-2-glycoprotein 1 (Apo-H) were enriched with OxPAPC (both liposome-based and multiwell plate-based pull-down) as well as with heparin. Both proteins showed significant masking activity at concentrations of 10 μg/ml (Apo-D) and 20 μg/ml (Apo-H) in the presence of 10 mg/ml BSA. (Fig. 7A). These concentrations are approximately 6-fold lower than reported physiological concentrations of Apo-D (70) and Apo-H (71) in human plasma. Apolipoprotein M (Apo-M) which was also identified as OxPAPC- and heparin-binding, statistically significantly reduced mAb 509 binding at 5 μg/ml (Fig. 7B). This concentration is about 4-fold lower than the physiological concentration of Apo-M in plasma (72).

Pulmonary surfactant-associated protein B (SP-B), identified in OxPAPC pull-down approaches but not found to interact with heparin, exhibited masking activity comparable to physiological concentrations of PAF-AH (Fig. 7C).

Antithrombin-III (ATIII), which is well known for its heparin-binding properties, was enriched with OxPAPC-containing liposomes and demonstrated masking activity at concentrations 20-fold lower than physiological plasma levels (73) (Fig. 7D). It has to be emphasized that all tested proteins (concentrations from 5 to 20 μg/ml) were incubated with OxLDL in the presence of 10,000 μg/ml BSA but nevertheless demonstrated significant masking activity.

## Discussion

A large body of data shows that OxPLs, similarly to other OSEs, can induce inflammation via binding to scavenger and other pattern recognition receptors, as well as antibodies, Fc receptors, and complement components (29). An important mechanism neutralizing OSEs is a deposition of circulating proteins on the surface of protein-OSE adducts (35). In order to distinguish this phenomenon from opsonization by complement C3b, which covalently binds foreign molecules and targets them for phagocytosis, non-covalent binding of plasma proteins to OSEs will be referred to as “masking”, which prevents recognition of OSEs by pro-inflammatory proteins and receptors. This mode of action can be compared, for example, with the action of blocking anti-VEGF antibodies, which bind the cytokine and prevent its interaction with cellular receptors. Although the biological relevance and therapeutic potential of antibody-dependent and -independent masking of OxPLs have been documented (15, 34), the details of this process are incompletely characterized. For example, while in mice antibodies recognizing phosphorylcholine group (PC) in bacterial polysaccharides or OxPCs are germline-encoded natural antibodies of the IgM class (74), in humans the anti-PC repertoire contains both IgM and IgG-switched immunoglobulins, most of which were characterized by extensive somatic mutations within their CDRs typical of a common antigen-driven immune response (75). Furthermore, very little is known about non-immunoglobulin plasma proteins capable of masking OxPLs. Although three such proteins have been identified previously (30, 34, 40), a big picture of the abundance and relative activity of circulating OxPL-masking proteins is missing. In this study, we used pull-down proteomics to identify circulating human proteins preferentially binding to OxPAPC-containing liposomes as compared to liposomes with non-oxidized PAPC.

We found that OxPAPC-containing liposomes preferentially bound 198 proteins as compared to PAPC. From these, 48 were immunoglobulin chains. All three major immunoglobulin classes (IgM, IgG and IgA) were enriched, including a variety of immunoglobulin gene families known to be common in OxPAPC-binding immunoglobulins. These include for example *IGHV3-7* and *IGKV4-1* genes known to be overrepresented in PC-reactive IgM + memory, IgM + CD27 + CD43+, and IgG-switched B cells (75) However, many *IGHV* genes that we found in OxPAPC-enriched antibodies were not present in the PC-reactive stereotyped B cell receptor (BCR) repertoire (75), indicating either that oxPAPC- and PC-reactive immunoglobulins may bind to different epitopes or conformational states of the PC headgroup, or that PC-specific BCR repertoires differ from secreted antibodies. This notion may be supported by the fact that the IgG pool enriched by OxPAPC-liposomes did not contain IgG2 immunoglobulins, whereas PC-reactive IgG + memory B cells preferentially expressed IgG2-type BCRs (75). Interestingly, a considerable fraction of OxPAPC-enriched immunoglobulins used the *IGHD*-proximal *IGHV* gene segments *IGHV1-3*, *IGHV1-8* or *IGHV2-5*, a finding that is reminiscent of fetal mouse B1 cells that preferentially use *IGHD*-proximal *IGHV* gene segments for producing prototypical natural IgM antibodies (76, 77), suggesting that these OxPAPC-binding immunoglobulins may derive from a human B1 cell equivalent. Further support for this hypothesis comes from a recent study showing that *IGHV1-8* was the most frequently used *IGHV* gene segment in human prenatal B1 cells (78). Together, these data indicate that immunoglobulins purified by OxPAPC-liposomes may show some characteristics of natural antibodies and point to a human B1 cell origin, however, the lack of detailed sequence information prevents final conclusions.

In addition to antibodies, also multiple circulating non-immunoglobulin proteins were enriched in eluates from OxPAPC liposomes. In agreement with previous data on OxPL-binding proteins (30, 34, 40), we observed significantly increased binding of CFH and Apo-M, also CRP was elevated, although it did not reach statistical significance (*P* < 0.08). In addition to these three known OxPL-binding proteins, multiple other high- and low-abundance proteins belonging to different structural and functional families have been identified (supplemental File S1).

The most significantly enriched GO term in the functional annotation of OxPAPC-binding proteins was “glycosaminoglycan binding”. In agreement with this finding, about two-third of OxPAPC-binding proteins were also adsorbed on heparin-Sepharose, which points to the importance of charged residues on circulating proteins for binding to OxPLs. Interestingly, electrostatic interactions may be a common theme in recognition of OSEs by binding partners as has been shown for the interaction of CFH with protein-MDA adducts (79), MCP-1 with OxLDL and OxPAPC (80) as well as OxLDL with oxidized low-density lipoprotein receptor 1 (81) and OxLDL and OxPAPC with scavenger receptor CD36 (82). It has been suggested that the binding of OxPAPC to CD36 is explained by the interaction of a receptor domain containing two conserved lysins with oxidatively truncated OxPCs containing omega-terminal carboxylic groups (82). Further studies are required to establish if negatively charged OxPCs generated by oxidation are important for binding of circulating masking proteins with OxPAPC.

In order to test if newly identified plasma proteins indeed can shield OxPLs, we used an immune “masking” assay. Because the adsorption of plasma proteins to OSEs-modified proteins is the central event in masking, we developed a method mimicking this process and analyzing formation of a protein “barrier” isolating OSEs from binding to other macromolecules (47). OxLDL immobilized in 96-well plates was used as a “natural” carrier of OSEs (in this case OxPLs), while monoclonal antibodies recognizing oxidized phosphatidylcholine (OxPC) or oxidized phosphatidylethanolamine (OxPE) were used as “detectors” showing if OxPLs on OxLDL are physically accessible to external soluble proteins. We have found that pre-treatment of immobilized OxLDL with diluted plasma strongly reduced the subsequent binding of monoclonal antibodies recognizing OxPC and OxPE. Selective removal of immunoglobulin classes showed an important role of IgM in the masking activity of plasma. On the other hand, the masking activity was completely inhibited by adding heparin to plasma and was removed from plasma by adsorption on heparin-Sepharose, which did not bind the bulk of IgM. Thus, our data suggest that both plasma immunoglobulins, mainly of class M, as well as non-immunoglobulin heparin-binding proteins, act in concert to mask OxPLs exposed on the surface of the OxLDL particle.

We tested several individual plasma proteins for their ability to mask OxLDL in our immune assay. As expected, both commercial and in-house purified human pooled IgM demonstrated masking activity. In contrast, complement factors triggering classical and alternative pathways (C1q, C3, and factor B) bound to OxPAPC but did not mask OxPLs. This was different from the complement pathway inhibitor, CFH, which both bound to OxPAPC and reduced the binding of anti-OxPL antibodies. These data suggest that binding to OxPLs is necessary but not sufficient for the masking activity.

Apo-M, Apo-H, and Apo-D, identified to be OxPAPC- and heparin-binding in our study, showed significant masking activity. Apo-M has been reported to interact with OxPLs, likely due to its lipocalin structure (40); overexpression of this protein in LDL-receptor knockout mice resulted in protection from atherosclerosis (83). Apo-H has been found to form complexes with circulating OxLDL; this complex formation could be inhibited by heparin (84, 85). Apo-D, also classified as a lipocalin, has not been tested for the ability to directly interact with OxPLs, but was implicated in the control of lipid peroxidation-dependent pathology (86). Although indirect, these data are in agreement with the protective role of these proteins in OSE-driven pathologies.

Interestingly, the well-known heparin-binding protein Antithrombin-III was enriched with OxPAPC-containing liposomes and exhibited masking activity at concentrations 20-fold lower than physiological plasma levels. This finding further suggests that OxPL- and heparin-binding proteins share common structural features crucial for binding and thus allows hypothesizing that ATIII and other proteins binding highly sulfated IdoA-rich GAGs may play an as yet unrecognized role in protection against OSEs.

To summarize, published data and results of this study show that blood plasma attenuates toxic and proinflammatory effects of OxPLs on blood and vessel wall cells and that enzymatic degradation by PLA2 and opsonization by natural antibodies are important mechanisms but cannot explain the entire detoxifying capacity of human plasma. Our data provide additional support for the importance of circulating non-immunoglobulin proteins in the neutralization of OxPLs. Importantly, newly identified OxPAPC-binding proteins at physiological concentrations demonstrated masking activity comparable to or exceeding that of CFH and Apo-M. Our findings support the notion that the masking of OxPLs by non-immunoglobulin plasma proteins is mediated by multiple binding proteins rather than a few proteins having high masking activity.

## Data availability

The mass spectrometry proteomics data have been deposited to the ProteomeXchange Consortium via the PRIDE (87) partner repository with the dataset identifiers PXD055177 (liposome pull-down) and PXD055179 (heparin pull-down). Further inquiries can be directed to the corresponding authors.

## Supplemental data

This article contains supplemental data.

## Conflict of interest

The authors declare that they have no conflicts of interest with the contents of this article.
