# Supplementary material for: Identification of plasma proteins binding oxidized phospholipids using pull-down proteomics and OxLDL masking assay
Source: J Lipid Res. 2024 Nov 19;66(1):100704. doi: 10.1016/j.jlr.2024.100704 (PMC11696850; doi:10.1016/j.jlr.2024.100704)
Supplement: Supplemental Figures [file mmc1.docx]

**Supplemental Figures**


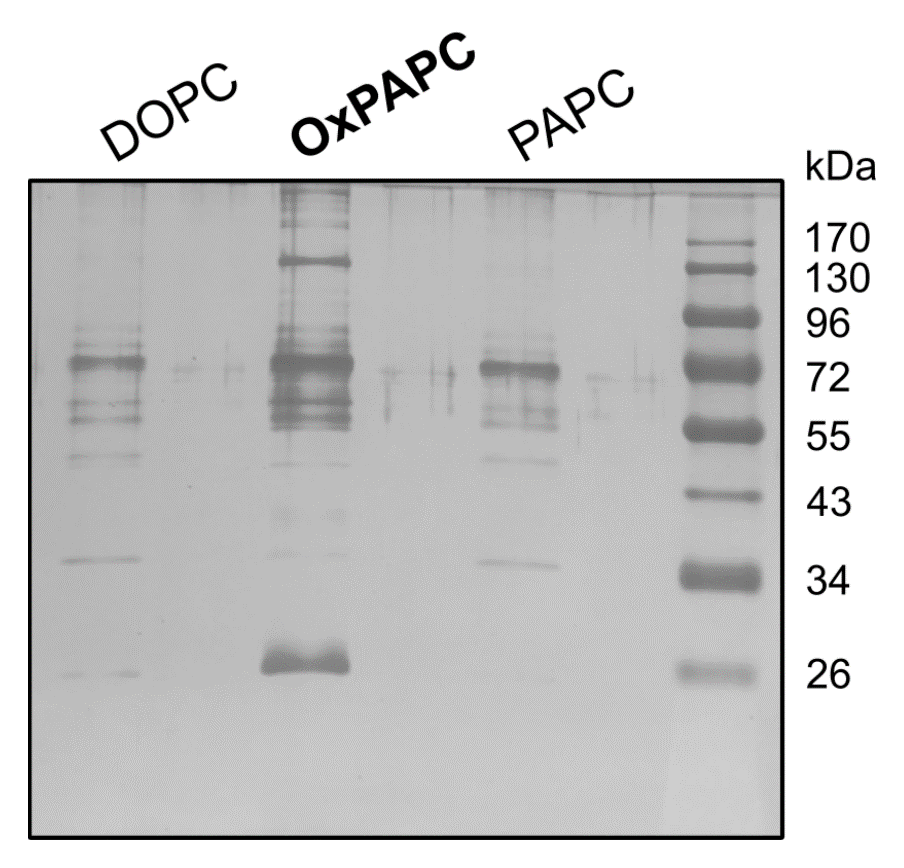


**Supplemental Figure S1.** Silver stain of proteins enriched by 96-well plate pull-down using OxPAPC, PAPC or DOPC as baits for plasma proteins.


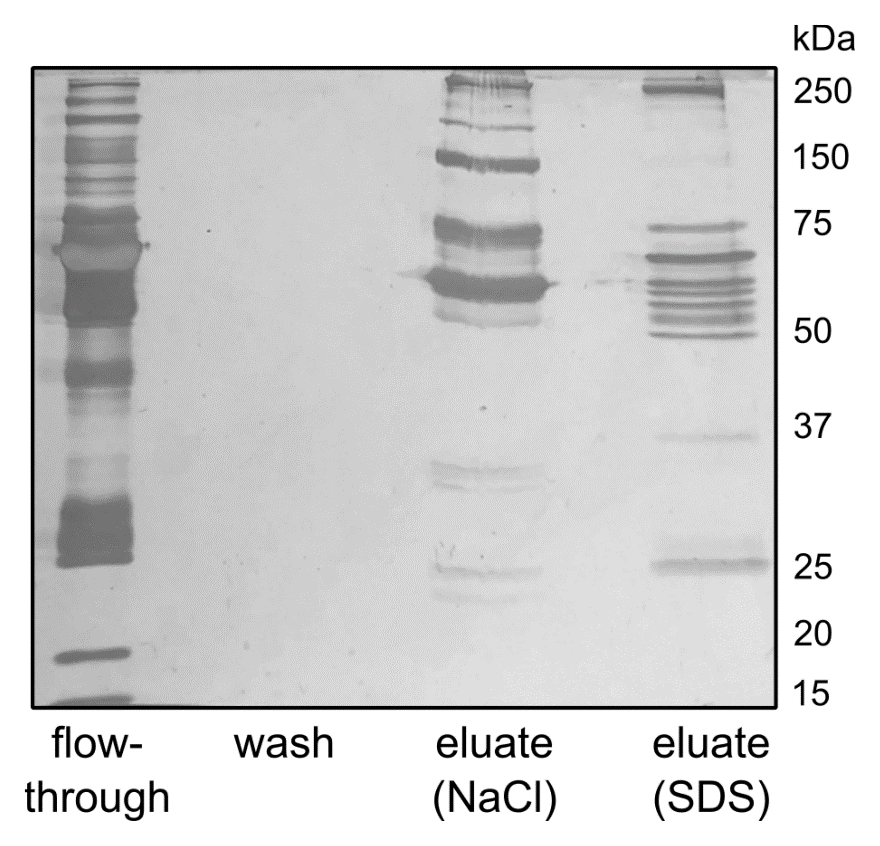


**Supplemental Figure S2.** Silver stain of proteins from heparin pull-down.


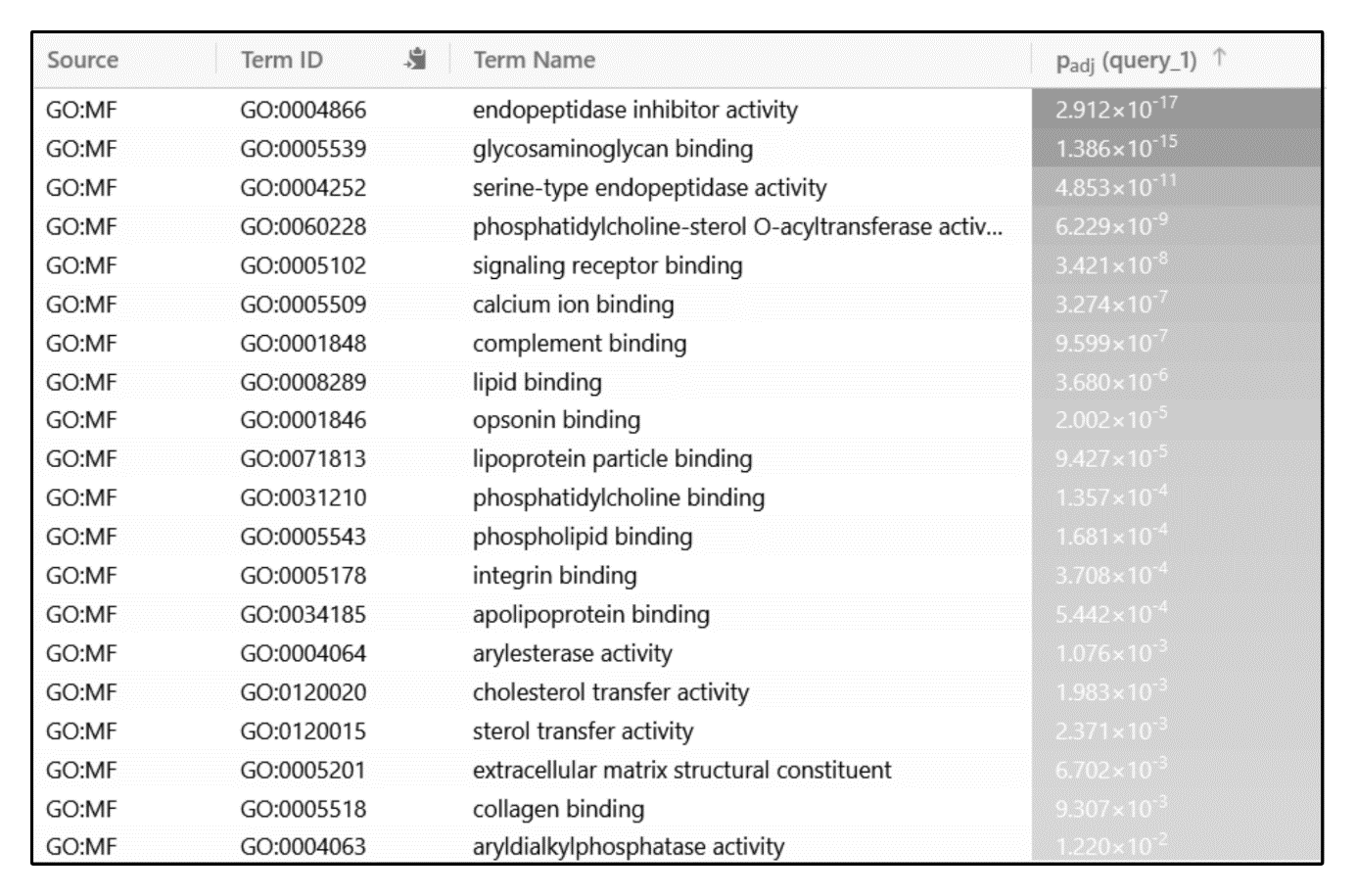


**Supplemental Figure S3.** Functional enrichment analysis of the overlap (129 proteins) between heparin- and OxPAPC-liposome pull-down proteomics, excluding immunoglobulins.


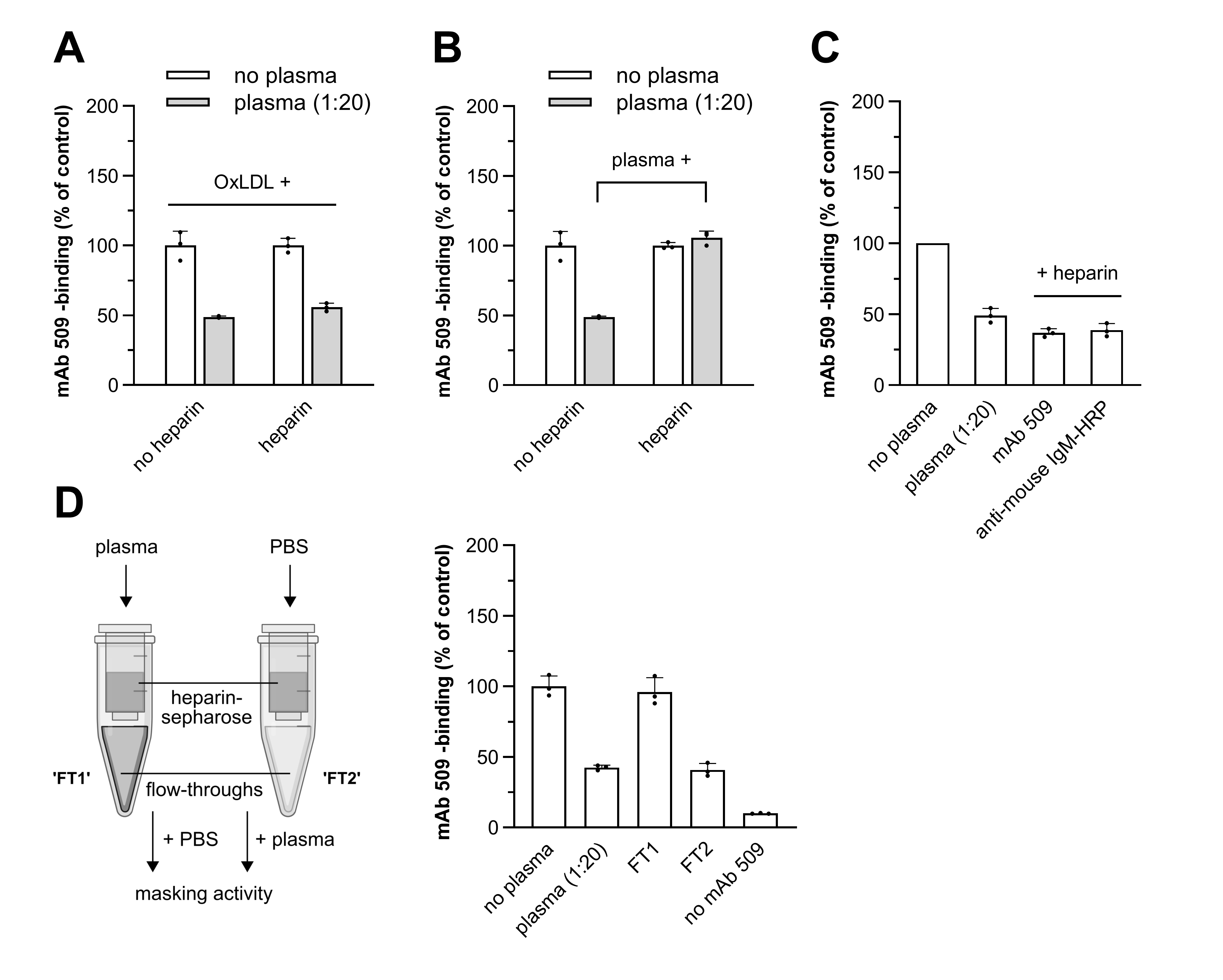


**Supplemental Figure S4.** Heparin inhibits OxLDL-masking activity through interaction with plasma proteins but not with OxLDL or primary or secondary antibodies.

A-D: Masking assay and chromatography were performed as described in the “Methods” section except that heparin was added at different incubation steps as described below. Plasma was diluted 1:20 in PBSE/BSA (10 mg/ml). Data are presented as means +/- SD.

A: OxLDL-coated plate was pre-incubated with or without heparin (20 µg/ml in PBSE) for 5 minutes at room t° followed by washing and incubation with blood plasma for 1 hour at 37°C. OxPLs were detected using mAb 509 and anti-mouse IgM-HRP.

B: OxLDL-coated plate was incubated with or without plasma diluted 1:20 in PBSE/BSA (10 mg/ml) in the presence or absence of heparin (20 µg/ml), followed by staining with mAb 509.

C: OxLDL-coated plate was incubated with or without plasma diluted 1:20 in PBSE/BSA (10 mg/ml), followed by detection by mAb 509 and anti-mouse IgM-HRP in the presence or absence of heparin (20 µg/ml in PBSE/BSA (10 mg/ml)).

Note, that the reversal of masking activity was observed when heparin was incubated with blood plasma (B) but not with OxLDL (A) or antibodies (C).

D: Experimental setup for testing potential heparin leakage from heparin-Sepharose into flow-through fraction (left) and subsequent testing of masking activity (right). Blood plasma (1:5 in PBS) or PBS were applied to columns containing heparin-Sepharose. Flow-through of plasma (FT1) was diluted 1:20 with PBS and tested for masking activity. Flow-through of PBS (FT2) was mixed with plasma and also tested for masking activity. Full masking activity of plasma in the presence of FT2 indicate that the loss of masking activity of plasma after heparin chromatography (FT1) cannot be explained by heparin leakage leading to contamination of plasma samples.


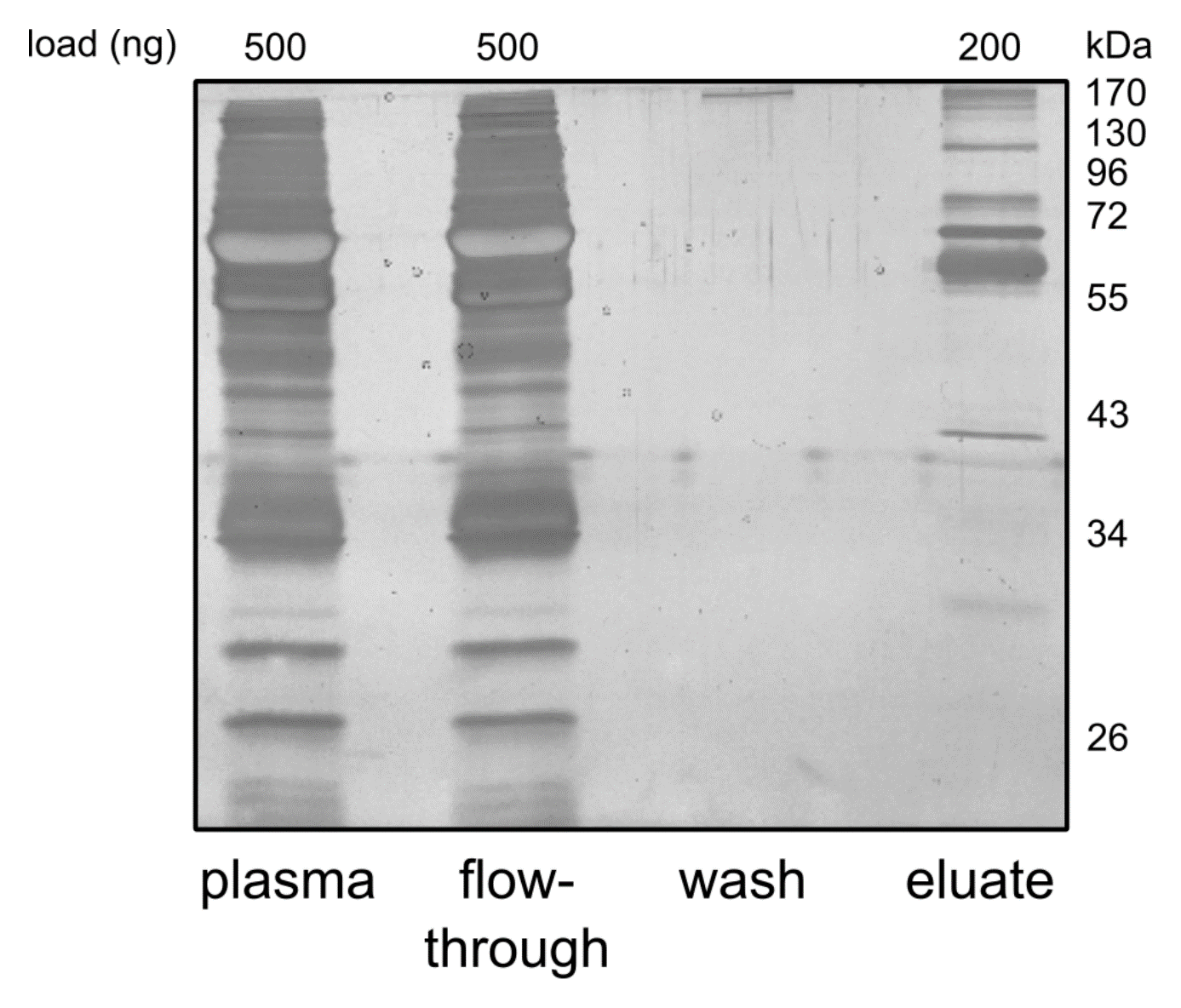


**Supplemental Figure S5.** Silver-stained SDS-PAGE of flow-through and eluate from heparin chromatography for the masking assay.


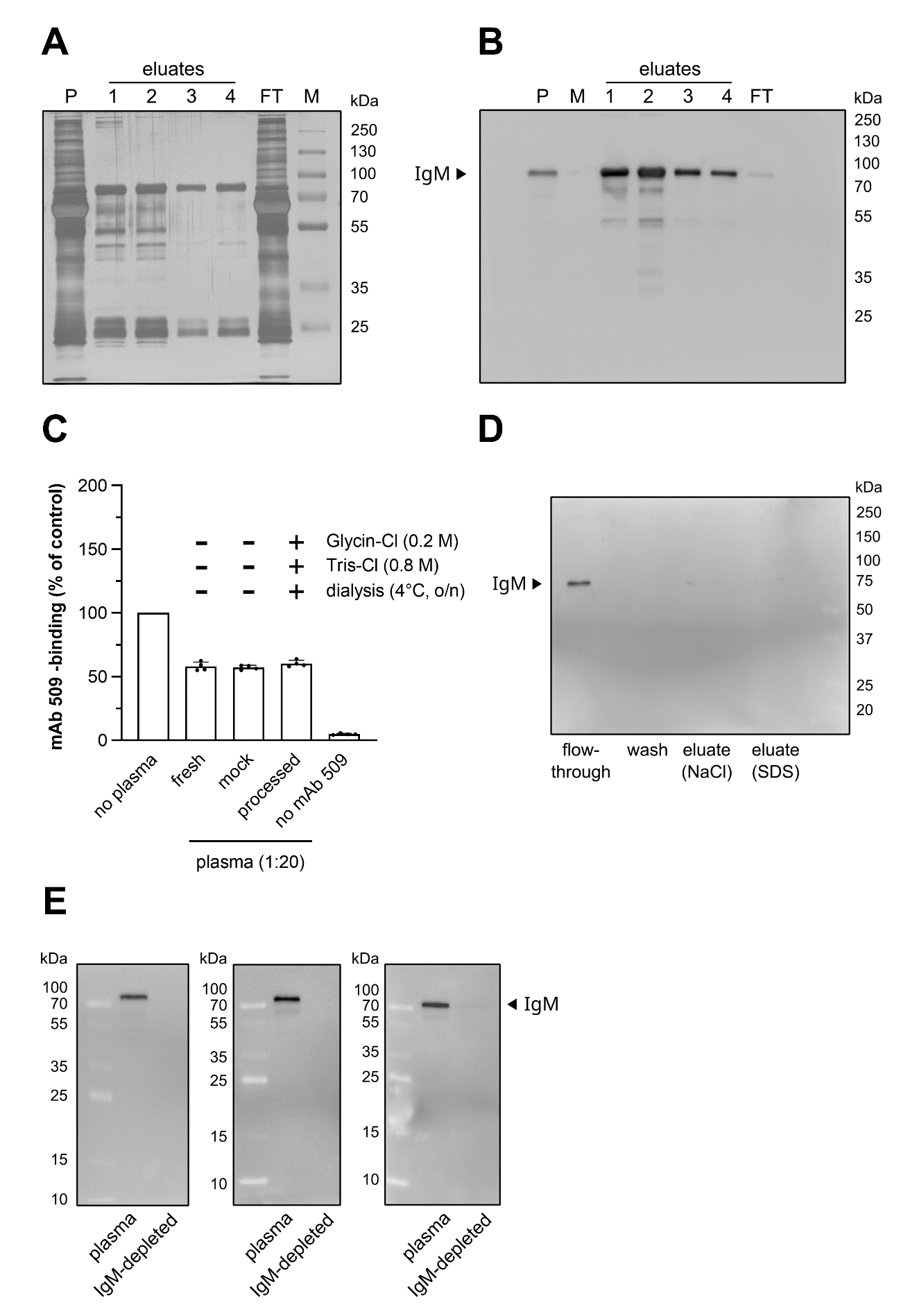


**Supplemental Figure S6.** Chromatographic procedures of IgM purification used in this study do not inactivate masking mechanisms.

A: Silver-stained SDS-PAGE of eluates containing purified IgM (E1-E4). Heavy (~80 kDa) and light chains (~20 kDa) are visible.

B: Heavy chains of purified IgMs were visualized by western blotting.

C: The masking capacity of plasma is not affected by the purification procedure, including steps of acidification (Glycine-HCl, 0.2 M), neutralization (Tris-Cl, 0.8 M) and dialysis. Processed plasma shows no significant changes in its capacity to mask OxLDL from mAb 509 compared to fresh blood plasma sample and mock-treated plasma. Mock-treated plasma was incubated for the same duration and temperature as the processed plasma.

D: IgM does not bind to heparin-Sepharose. Flow-through and eluates obtained using heparin-Sepharose were analyzed by western blotting for the presence of IgM. Note that IgM is almost entirely contained in the flow-through fraction.

E: Western blots of IgM-depleted fractions obtained in different experiments showing high efficacy and reproducibility of procedures used to deplete plasma from endogenous IgMs.


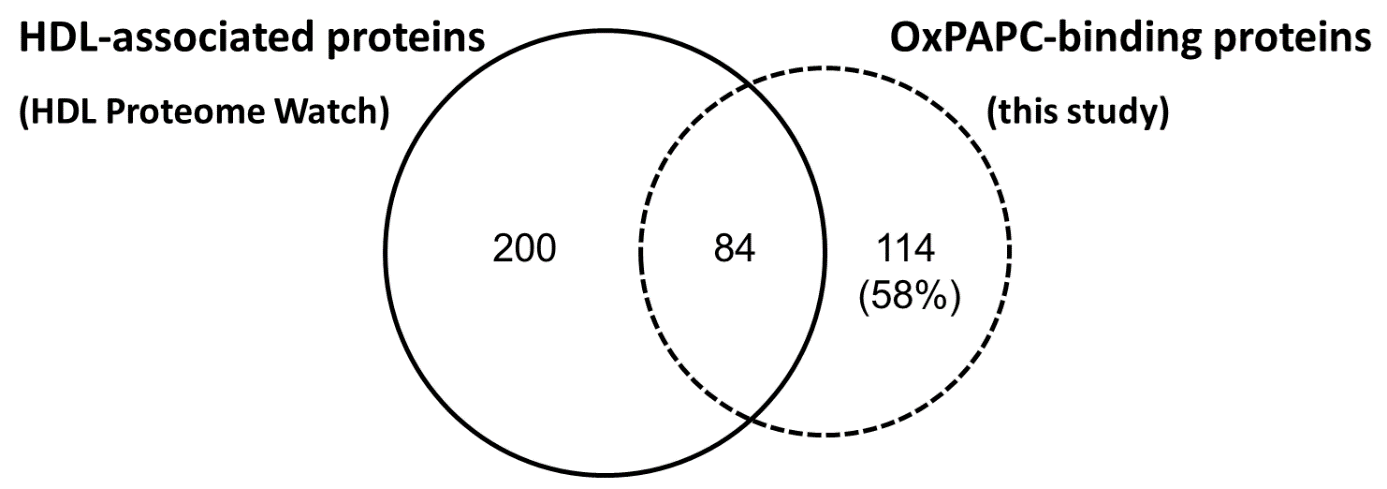


**Supplemental Figure S7.** Overlap between OxPAPC-binding proteins identified in this study and 284 proteins from HDL Proteome Watch database (appearing in 3 from 51 studies surveyed).
